# Safety and immunogenicity of a SARS-CoV-2 inactivated vaccine in patients with chronic hepatitis B virus infection

**DOI:** 10.1038/s41423-021-00795-5

**Published:** 2021-11-15

**Authors:** Tiandan Xiang, Boyun Liang, Hua Wang, Xufeng Quan, Shengsong He, Helong Zhou, Yongwen He, Dongliang Yang, Baoju Wang, Xin Zheng

**Affiliations:** 1 Department of Infectious Diseases, Union Hospital, Tongji Medical College, Huazhong University of Science and Technology, Wuhan, China; 2 Joint International Laboratory of Infection and Immunity, Huazhong University of Science and Technology, Wuhan, China

**Keywords:** Hepatitis B, Inactivated vaccines, Antibodies

Coronavirus disease 2019 (COVID-19), caused by SARS-CoV-2 infection, has become a major global public health threat. Although significant advances have been made in developing and applying different vaccines in clinical trials [1, 2], data are limited on the safety and efficacy of the inactivated vaccine in patients with chronic liver disease [3]. Recent studies have preliminarily described the safety and immunogenicity of SARS-CoV-2 vaccines in patients with nonalcoholic fatty liver disease and in liver transplant recipients [4, 5]. However, to date, there is no detailed information on the SARS-CoV-2 inactivated vaccine in patients with chronic hepatitis B (CHB) infection. It has been reported that CHB patients have impaired immune systems [6]. Hence, whether immunocompromised CHB patients within the different clinical stages can be safely vaccinated with the various types of SARS-CoV-2 vaccines and produce an effective immune response remains unclear. Our study aims to provide a comprehensive analysis from different clinical dimensions to characterize the safety and immunogenicity of SARS-CoV-2 inactivated vaccines (BBIBP-CorV, CoronaVac, or WIBP-CorV) within this specific patient population.

A total of 284 CHB patients who were unvaccinated (*n* = 81) or had completed the first (*n* = 54) or second dose (*n* = 149) of the vaccines were enrolled from March 23, 2021, to September 10, 2021 (Table S1). The median time post-vaccination was 33 (IQR, 24–48) days among the 149 completely vaccinated patients. Safety was evaluated by determined the overall incidence of adverse reactions via a standardized questionnaire. Moreover, plasma samples were examined for IgG antibodies against the receptor-binding domain (RBD) of the SARS-CoV-2 spike protein (anti-S-RBD-IgG) and for neutralizing antibodies (NAbs). The complete methods regarding the study design and the statistical analysis are available in the Supplementary methods section.

The adverse reaction data were first analyzed in 149 completely vaccinated CHB patients. The overall incidence of adverse reactions within 7 days was 30.2% (Table S2), which was similar to that found in the phase 3 trials of CoronaVac in Turkey [2]. The most common side effect was injection-site pain (25.5%, 38/149), followed by drowsiness (3%, 3/149); only one patient reported fever on the first day after vaccination. Almost all of the adverse reactions were mild and self-resolved within a few days after vaccination. Serious side effects were not observed even in 20 CHB patients with abnormal alanine aminotransferase levels [61.5 (43–129) U/L] or 10 patients with compensated liver cirrhosis. The results demonstrated that SARS-CoV-2 inactivated vaccines had a favorable safety profile in CHB patients. Given that previous studies have shown an increased risk of progression to severe disease in COVID-19 patients with cirrhosis [7], the benefit of vaccination in compensated cirrhotic patients still outweighs the vaccine-related risk.

Next, we determined the immunogenicity of CHB patients who completed the two doses of the vaccination regimen. The seropositivity for anti-S-RBD-IgG and NAbs was 87.25% and 74.5%, respectively (Fig. 1A). The anti-S-RBD-IgG seropositivity of CHB vaccine recipients was similar to that in a clinical trial of CoronaVac in Turkey (89.7%) but much higher than the reported recently seropositivity of IgG antibodies to the spike protein (76%) in patients with chronic liver disease [5]. Both anti-S-RBD-IgG and NAb levels increased significantly to a higher level after completing the vaccination regimen (Fig. 1B, C, *P* < 0.0001). This finding indicates that SARS-CoV-2 inactivated vaccines can elicit an optimal antibody response even though some CHB patients may have pre-existing compromised immune function.

**Fig. 1:**
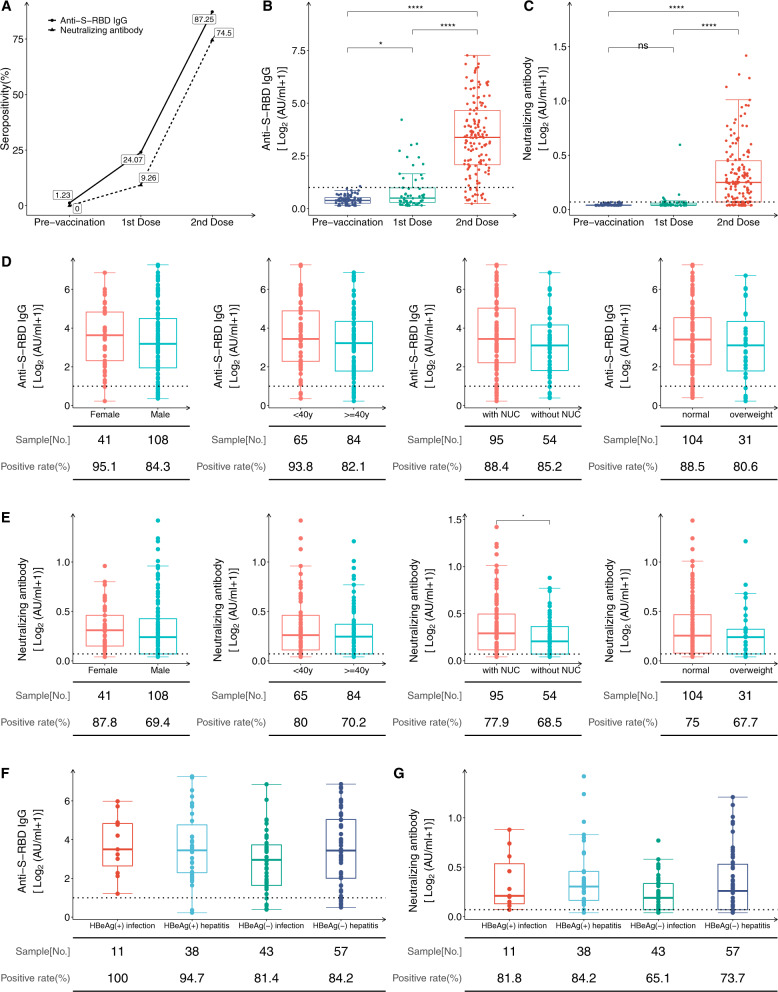
Antibody responses following immunization with the inactivated vaccine in CHB patients. **A** The seropositivity of anti-S-RBD-IgG and NAbs in CHB patients. **B**, **C** Kinetics of the anti-S-RBD-IgG and NAb titers in vaccine-induced sera at different time points in CHB patients. Prevaccination, *n* = 81; first dose, *n* = 54; second dose, *n* = 149. **D**, **E** The comparison of anti-S-RBD-IgG and NAb titers stratified according to sex, age, nucleos(t)ide analog (NUC) therapy, and BMI (overweight: BMI ≥ 25; 14 patients had unavailable BMI values). **F**, **G** Comparison of anti-S-RBD-IgG (**F**) and NAb titers (**G**) in HBeAg^+^ chronic infection, HBeAg^+^ chronic hepatitis, HBeAg^−^ chronic infection, and HBeAg^−^ chronic hepatitis individuals [9]. Sample numbers and positive rates are shown underneath. *P* values were determined using a Mann–Whitney U test or a Kruskal–Wallis test followed by Dunn’s multiple comparisons test for antibody titers and Fisher’s exact test for seropositivity. The horizontal dotted line represents the cutoff value. ns: no significance, *p < 0.05, ****p < 0.0001

The seropositivity and antibody titers in CHB patients were further compared according to sex, age, antiviral therapy, and body mass index stratification (Fig. 1D, E). We found that younger patients (<40 y) had higher seropositivity for anti-S-RBD-IgG (*P* < 0.05), and female patients exhibited increased seropositivity for NAbs (*P* < 0.05). Recent clinical trials have also reported a similar trend: younger individuals and female vaccine recipients exhibited stronger humoral immune responses to vaccination [2]. Interestingly, the patients undergoing nucleos(t)ide analog therapy had a significantly higher NAb titer than those who were not (*P* < 0.05) (Fig. 1D, E). Long-term antiviral therapy can inhibit viral replication and facilitate the restoration of the impaired immune system by recovering the function of circulating dendritic cells, natural killer cells, or T cells, particularly nucleotide analogs that can induce the production of IFN-λ3 [6, 8]. These factors may account for the higher antibody titer in patients with antiviral therapy. Given that nucleos(t)ide analog therapy does not affect vaccine-induced immune responses, it should be continuously administered during vaccination to avoid negatively impacting CHB treatment.

Finally, we compared the antibody responses among the CHB patients in the various clinical stages of infection. The CHB participants were divided into four groups according to the “EASL 2017 Clinical Practice Guidelines on the Management of Hepatitis B Virus Infection” [9]: (I) HBeAg-positive chronic HBV infection, (II) HBeAg-positive chronic hepatitis B, (III) HBeAg-negative chronic HBV infection, and (IV) HBeAg-negative chronic hepatitis B. There was no significant difference in seropositivity or antibody titers among the four groups constituting the 149 CHB patients (Fig. 1F, G), suggesting the general applicability of the inactivated vaccines within this patient population.

Altogether, our study reveals that SARS-CoV-2 inactivated vaccines achieve a favorable safety profile and efficient immunogenicity in patients with CHB in real-world vaccination scenarios. The results are encouraging despite some patients not being vaccinated following the standard dose interval time in clinical trials or the two dosages of the inactivated vaccine not being from the same manufacturer.

## Supplementary information

Supplementary Methods

Supplementary Table
