# Supplementary material for: Safety and immunogenicity of a SARS-CoV-2 inactivated vaccine in patients with chronic hepatitis B virus infection
Source: Cell Mol Immunol. 2021 Nov 15;18(12):2679–81. doi: 10.1038/s41423-021-00795-5 (PMC8591435; doi:10.1038/s41423-021-00795-5)
Supplement: Supplementary file 1 — Supplementary Methods [file 41423_2021_795_MOESM1_ESM.docx]

Supplementary File 1

**Methods**

***Study design and participant information***

This study was done at Wuhan Union Hospital, Tongji Medical College, Huazhong University of Science and Technology in Wuhan, Hubei, China. We enrolled patients with chronic hepatitis B (CHB) who are pre-vaccinated or have completed the first or second dose of SARS-CoV-2 inactivated vaccines ( (BBIBP-CorV, CoronaVac, or WIBP-CorV)) from March 23, 2021 to September 10, 2021. All participants were older than 18 years-old, without a known history of SARS-CoV-2 infection. The exclusion criteria were as follows: (1) those who had a cold, tooth extraction, or tonsils in the past week; (2) those with autoimmune diseases or who are taking hormones and immunosuppressive drugs; (3) Patients with severe underlying diseases.

The diagnostic criteria for chronic HBV infection were: HBsAg or HBV DNA positive for at least 6 months. According to the “EASL 2017 Clinical Practice Guidelines on the management of hepatitis B virus infection ”[1], all CHB participants were divided into four groups taking into account the presence of HBeAg, HBV DNA levels, alanine aminotransferase (ALT) values: (I) HBeAg-positive chronic HBV infection, (II) HBeAg-positive chronic hepatitis B, (III) HBeAg-negative chronic HBV infection, (IV) HBeAg-negative chronic hepatitis B.

Clinical data on anti-HBV treatment, HBV serologic biomarkers, and liver function test results were extracted from electronic medical records prior to the date of the first vaccination. Abnormal ALT test was defined values greater than the upper limit of normal (40U/L). The presence or absence of cirrhosis was determined by clinical evidence combined with liver imaging examination. The study was approved by the Ethics Committee of Wuhan Union Hospital, Tongji Medical College, Huazhong University of Science and Technology. It has been registered in the Chinese clinical trial registry (ChiCTR2100048840).

***Vaccine safety investigation***

Adverse reactions after vaccination were collected by filling a standard questionnaire under the guidance of professional physicians, including local (pain, swelling, and induration) or systematic reactions (fever, fatigue, drowsiness, headache, dizziness, and myalgia). The primary safety outcome was the overall incidence of adverse events within 7 days.

***SARS-CoV-2 antibodies detection***

Plasma samples were collected at least two weeks following each episode of vaccination to detect IgG antibody against receptor binding domain (RBD) of SARS-CoV-2 spike protein (anti-S-RBD-IgG) and neutralizing antibodies (NAbs) using *capture chemiluminescence immunoassays* by MAGLUMI™ X8 (Snibe, Shenzhen, China) according to the manufacturer’s instruction. The kit reported that anti-S-RBD-IgG tests have 100% sensitivity（≥15 days post symptom onset ）and 99.6% specificity for the diagnosis of COVID-19, while NAbs tests have 100% sensitivity and 100%% specificity respectively. The cut-off value was 1 AU/mL for Anti-S-RBD-IgG and 0.05 AU/mL for NAbs.

***Statistical analysis***

Categorical variables were expressed as percentage, while continuous variables were expressed as mean ± SD or median (IQR) as appropriate. Chi-square test or Fisher's exact test was used to assess the significance between groups for categorical variables. The Kruskal-Wallis test (three or more groups) or Mann–Whitney U test (two groups) were used to assess the significant differences for continuous variables. Statistical significance was determined by SPSS version 25.0 (IBM, Chicago, IL, USA). All reported P values were two-tailed, and P <0.05 was considered statistically significant.

**References:**

1. EASL 2017 Clinical Practice Guidelines on the management of hepatitis B virus infection. J Hepatol 2017;67: 370-98.
