# Supplementary material for: Safety and immunogenicity of a SARS-CoV-2 inactivated vaccine in patients with chronic hepatitis B virus infection
Source: Cell Mol Immunol. 2021 Nov 15;18(12):2679–81. doi: 10.1038/s41423-021-00795-5 (PMC8591435; doi:10.1038/s41423-021-00795-5)
Supplement: Supplementary file 2 — Supplementary Table [file 41423_2021_795_MOESM2_ESM.docx]

**Supplementary File 2**

**Table S1. Clinical characteristics of chronic hepatitis B (CHB) patients**

| **Characteristics** | **Pre-vaccination (N=81)** | **1^st^ dose**  **(N=54)** | **2^nd^ dose**  **(N=149)** |
| --- | --- | --- | --- |
| **Age, [median (IQR)]** | **38 (34-49)** | **37.5 (34-44)** | **41 (33-49)** |
| <40y, [n (%)] | 44 (54.3) | 29 (53.7) | 65 (43.6) |
| ≥40y, [n (%)] | 37 (45.7) | 25 (46.3) | 84 (56.4) |
| **Sex, [n (%)]** |  |  |  |
| Male | 56 (69.1) | 32 (59.2) | 108 (72.5) |
| Female | 25 (30.9) | 22 (40.7) | 41 (27.5) |
| **Antiviral treatment, [n (%)]** |  |  |  |
| With NUC | 61 (75.3) | 42 (77.8) | 95 (63.8) |
| Without NUC | 20 (24.7) | 12 (22.2) | 54 (36.2) |
| **Abnormal ALT, [n (%)]** |  |  |  |
| ALT＞40U/L | 11 (13.6) | 6 (11.1) | 20 (13.4) |
| **Compensated cirrhosis [n (%)]** | 5 (6.2) | 2 (3.7) | 10 (6.7) |
| **HBV phase, [n (%)]** |  |  |  |
| HBeAg^+^ chronic HBV infection | 5 (6.2) | 1 (1.9) | 11 (7.4) |
| HBeAg^+^ chronic hepatitis B | 24 (29.6) | 20 (37.0) | 38 (25.5) |
| HBeAg^-^ chronic HBV infection | 15 (18.5) | 11 (20.4) | 43 (28.9) |
| HBeAg^-^ chronic hepatitis B | 37 (45.7) | 22 (40.7) | 57 (38.3) |
| **Comorbidities, [n (%)]** | 13 (16.0) | 7 (13.0) | 28 (18.8) |
| Hypertension | 3 (3.7) | 3 (5.6) | 9 (0.6) |
| Fatty liver disease | 6 (7.4) | 0 (0) | 11 (7.4) |
| Diabetes | 0 (0) | 1 (1.9) | 4 (2.7) |
| Cardiovascular disease | 0 (0) | 1 (1.9) | 2 (1.3) |
| COPD | 1 (1.2) | 0 (0) | 1 (0.7) |
| Cancer | 0 (0) | 0 (0) | 0 (0) |
| Other | 3 (3.7) | 3 (5.6) | 5 (3.4) |

Data are shown as median (interquartile range) or n (%). CHB，chronic hepatitis B；ALT, alanine aminotransferase; HBcAb, hepatitis B core antibody; HBeAg, hepatitis B e antigen; COPD, chronic obstructive pulmonary disease. NUC, nucleos(t)ide analogues therapy.

**Table S2. Safety of SARS-CoV-2 vaccination in patients with CHB.**

| **Characteristics** | **CHB（N=149）** |
| --- | --- |
| **Post-vaccination days**  **[median (IQR)]** | 33 (24-48) |
| **Total** **adverse reactions, [n (%)]** | 45 (30.2) |
| **Local reactions, [n (%)]** | 38 (25.5) |
| Injection-site pain | 38 (25.5) |
| Injection-site swelling | 0 (0) |
| Injection-site induration | 0 (0) |
| **Systematic reactions, [n (%)]** | 11 (7.4) |
| Fever | 1 (0.7) |
| Fatigue | 2 (1.3) |
| Drowsiness | 3 (2.0) |
| Headache | 0 (0) |
| Dizziness | 2 (1.3) |
| Nausea | 1 (0.7) |
| Myalgia | 1 (0.7) |
| Others | 2 (1.3) |
